# Incidence of juvenile idiopathic arthritis in Finland, 2000–2020

**DOI:** 10.1093/rheumatology/keae322

**Published:** 2024-06-10

**Authors:** Erika Uusitupa, Heidi Rahikkala, Sirja Sard, Tytti Pokka, Henri Salo, Johanna Kärki, Tuulikki Sokka-Isler, Maria Backström, Paula Vähäsalo

**Affiliations:** Department of Pediatrics, University of Turku, Turku, Finland; Department of Pediatrics, Turku University Hospital, Turku, Finland; Department of Pediatrics, Turku University Hospital, Turku, Finland; Research Unit of Clinical Medicine, University of Oulu, Oulu, Finland; Research Unit of Clinical Medicine, University of Oulu, Oulu, Finland; Department of Children and Adolescents, Oulu University Hospital, Oulu, Finland; Medical Research Center Oulu, Oulu University Hospital and University of Oulu, Oulu, Finland; Research Unit of Clinical Medicine, University of Oulu, Oulu, Finland; Research Service Unit, Oulu University Hospital, Oulu, Finland; Knowledge Brokers Department, Finnish Institute for Health and Welfare, Helsinki, Finland; Department of Children and Adolescents, Kanta-Häme Central Hospital, Wellbeing Services County of Kanta-Häme, Hämeenlinna, Finland; Wellbeing Services County of Central Finland/Hospital Nova of Central Finland, Jyväskylä and University of Eastern Finland, Kuopio, Finland; Research Unit of Clinical Medicine, University of Oulu, Oulu, Finland; Department of Pediatrics, Wellbeing Services County of Ostrobothnia, Vaasa, Finland; Research Unit of Clinical Medicine, University of Oulu, Oulu, Finland; Department of Children and Adolescents, Oulu University Hospital, Oulu, Finland; Medical Research Center Oulu, Oulu University Hospital and University of Oulu, Oulu, Finland

**Keywords:** juvenile idiopathic arthritis, incidence, epidemiology, children, register study

## Abstract

**Objective:**

Previous epidemiological data of JIA in Finland are from the turn of the millennium. We aimed to determine the recent annual incidence of JIA in several consecutive years in Finland and to explore the differences in incidence between sexes, age groups and regions.

**Methods:**

We analysed all children <16 years of age who met the ILAR classification criteria for JIA. Cases from 2000–2020 were identified from two national registers: the Care Register for Health Care of the Finnish Institute for Health and Welfare and the Reimbursement Register containing medication data from the Social Insurance Institution of Finland; cases from 2016–2020 were identified from the Finnish Rheumatology Quality Register.

**Results:**

The incidence of JIA was 31.7 per 100 000 (95% CI 30.2, 33.1), according to the Care Register in 2000–2020 and peaked in 2010–2014. No considerable differences in incidence rates were observed among registers. In all age groups, incidence in girls was predominant compared with boys. The incidence in girls peaked at the ages of 2 years and 14–15 years. Decreasing incidence was observed among boys 0–3 years old during the entire study period, whereas increasing incidence was observed among teenage girls and boys 4–7 years old in 2000–2013.

**Conclusion:**

The incidence of JIA is not only very high with respect to that in other parts of the world but also higher than previously reported in Finland. The incidence varied by region and year but was not higher at the end than the beginning of the study period.

Rheumatology key messages

- The incidence of JIA in Finland is high, from a global perspective, 31.7 per 100 000.
- The annual incidence was not higher in the year 2020 than 2000.
- The incidence decreased among the youngest boys and increased among teenage girls until 2013.

## Introduction

Juvenile idiopathic arthritis (JIA) is an inflammatory disorder of unknown aetiology beginning before the age of 16 years and characterized by inflammation of at least one joint persisting for >6 weeks [1, 2]. JIA is divided into seven categories in the International League of Associations for Rheumatology (ILAR) classification system, according to clinical and immunologic characteristics and family history [3–5]. Although JIA is the most common rheumatic disease in childhood [2], it is relatively rare; therefore, long-term studies are needed to determine the disease burden and to plan healthcare resources.

In a systematic review of JIA prevalence and incidence using three classification systems for JIA—the American College of Rheumatology, the European Alliance of Associations for Rheumatology and ILAR—the incidence rates have been found to range widely, from 1.6–23 per 100 000 [2]. The variation depended on the study design and geographical location and was likely to have been influenced by environmental factors and genetics [6]. Since then, according to the ILAR classification, incidence rates in Europe have varied, according to reports of 3.2 per 100 000 in France in 2001 [7], 5.6 per 100 000 in the UK in 2000–2018 [8] and 6.9 per 100 000 in Spain in 2004–2006 [9]. Differences have also been observed within continents; for example, incidence rates of 8.5–17.8 per 100 000 in Canada [10, 11] and 10.3 per 100 000 in the USA [12]. Moreover, incidence rates vary among the Nordic countries: the incidence has been reported to be 7.1 per 100 000 in Iceland [13], 12.8–17.1 per 100 000 in Sweden [13, 14], 19.0–23.0 per 100 000 in Norway [13], and highest, 24.1 per 100 000, in Denmark [15].

In Finland, the most recent epidemiological studies of JIA were conducted at the turn of the millennium [16] and suggested that the incidence per 100 000 rose from 14 to 19.5–23 [17, 18], with regional variations [16, 17]. However, those studies were from single years or covered only part of Finland [13, 16–19].

The aim of this study was to assess the recent annual incidence of JIA in Finland among various patient groups during several consecutive years, examine possible epidemiological changes, and compare the incidence rates across hospital districts and JIA categories.

## Materials and methods

### Registers

Data were collected from three registers: the Care Register for Health Care of the Finnish Institute for Health and Welfare (Care Register), the Reimbursement Register of the Social Insurance Institution of Finland (SII register) and the Finnish Rheumatology Quality Register (FinRheuma), also maintained by the Finnish Institute for Health and Welfare. In Finland, nearly every patient with JIA is treated in the public healthcare system by a paediatrician with competence in paediatric rheumatology or a paediatric rheumatologist; therefore, the data in the Care Register and the FinRheuma register are reliable.

The Care Register includes statistics on outpatient visits and care periods in specialized somatic healthcare collected from the entire country. According to Finnish law regarding personal registers, service providers are required to contribute information to this register.

If treatment with disease-modifying antirheumatic drugs (DMARDs) is started at the time of the diagnosis, a medication reimbursement application is prepared by the rheumatologist. The reimbursement is granted by SII for DMARDs. In addition to granting reimbursements for patients with chronic illnesses, such as JIA, SII maintains a database of individuals, including the International Classification of Diseases (ICD) code of the diagnosis and the date of reimbursement.

FinRheuma was started in 2018, but information since the end of the 1990s has been imported into the register. The information involves clinical aspects of JIA, such as diagnoses, category, visits, disease activity parameters and medications. This study included FinRheuma data from eight hospital districts: four university hospitals (Oulu, Kuopio, Turku and Helsinki University Hospitals) and four central hospitals (Tavastia Proper, Päijänne Tavastia, Central Ostrobothnia and Vaasa Central Hospitals), where a paediatrician or paediatric rheumatologist separately confirmed the data from the patient medical records during the years 2016–2020. JIA categories in the FinRheuma data were determined according to the ICD-10 code at the last visit when the patient was still younger than 16 years or, if absent, the first ICD-10 code after the age of 16 years.

Almost all JIA patients in Finland are registered in all three registers. It is not possible that one patient is counted twice within the same register.

### Study design and study population

This population-based register study included all children under 16 years of age who had been diagnosed with JIA and registered in the Care, SII or FinRheuma registers (Table 1). The patients’ hospital districts were determined according to the place of residence at the time of diagnosis. Parents in seven families declined to provide information on the place of residence. These children were not included in the regional analyses. ICD-10 code M08.9, describing the diagnosis of early JIA, was included in the SII register, as reimbursement is often applied at an early stage, when the JIA category is yet unspecified. It is possible that adult rheumatologists have treated a small number of teenage JIA patients from the beginning of the disease and thus they received an incorrect diagnosis; for example, rheumatoid arthritis instead of JIA. Therefore, we included these adult ICD-codes in the Care Register (Table 1). The annual population size of children <16 years of age was derived from the public database of Statistics Finland, according to hospital district, sex and age group.

**Table 1.** Study registers, study period, inclusion criteria, number of patients with JIA and population size

Register	Care Register	SII register	FinRheuma
Time of data collection	1 January 2000 to 31 December 2020	1 January 2000 to 31 December 2020	1 January 2016 to 31 December 2020
ICD-10 codes included	M08.0, M08.1, M08.2, M08.3, M08.4, M07.0, M07.1, M07.2, M07.3, M09.0, M05.3, M05.8, M05.9, M06.0, M06.1, M06.4, M06.8, M06.9, M45.x, M46.1 and M46.9	M08, M08.0, M08.1, M08.2, M08.3, M08.4, M08.8, M08.9 and M09.0*L40.5	M08, M08.0, M08.1, M08.2, M08.3, M08.4, M08.8 and M09.0*L40.5
Inclusion criteria	Two or more visits with the above diagnosis codes at the age of 0–15.9 years. If JIA was diagnosed at the age of 15.0–15.9 years, the second visit with the diagnosis code was allowed in the following year, at the age of <17 years.	The first time the above diagnosis code was mentioned during the study period, at the age of 0.0–15.9 years.	A timepoint of diagnosis in the register, which was confirmed from patients’ medical records by a paediatrician or a paediatric rheumatologist in eight hospital districts. Age at diagnosis 0.0–15.9 years.
Number of patients	6386	5556	887
Population size per year, range	year 2000: 1 000 497 to year 2020: 921 356	year 2000: 1 000 497 to year 2020: 921 356	year 2016: 607 802 to year 2020: 596 465

Care Register: the Care Register of National Institute for Health and Welfare; SII register: the Reimbursement Register of Social Insurance Institution of Finland; FinRheuma: the Finnish Rheumatology Quality Register; ICD-10: International Classification of Diseases, 10th Revision; JIA: juvenile idiopathic arthritis.

### Ethics

This register-based study used data from the FinRheuma register, which is maintained by the Finnish Institute for Health and Welfare, which also granted approval for this study.

### Statistical analyses

The incidence rates with 95% confidence intervals (95% CIs) were calculated by division of the number of new JIA diagnoses by the total population at risk during the study period and expressed per 100 000 children younger than 16 years. The statistical differences in incidence rates across sexes, age groups and regions were tested with a standardized normal deviate test. Joinpoint regression analysis on a logarithmic scale was used to detect increasing or decreasing trends in incidence over follow-up time segments. The analysis started with a zero joinpoint model; subsequently, we tested whether a model with more joinpoints might be statistically significant. The number of joinpoints indicated how frequently the trends changed during the follow-up. The start and end points of each segment were calculated to achieve the best fit to the data. The annual percentage change (APC) was used to characterize trends in the incidence rate of JIA that change at a constant percentage per year in each time segment. The average annual percent change (AAPC) described the change in rate over a predetermined time segment in the study, over the entire follow-up period.

**Figure 1.**
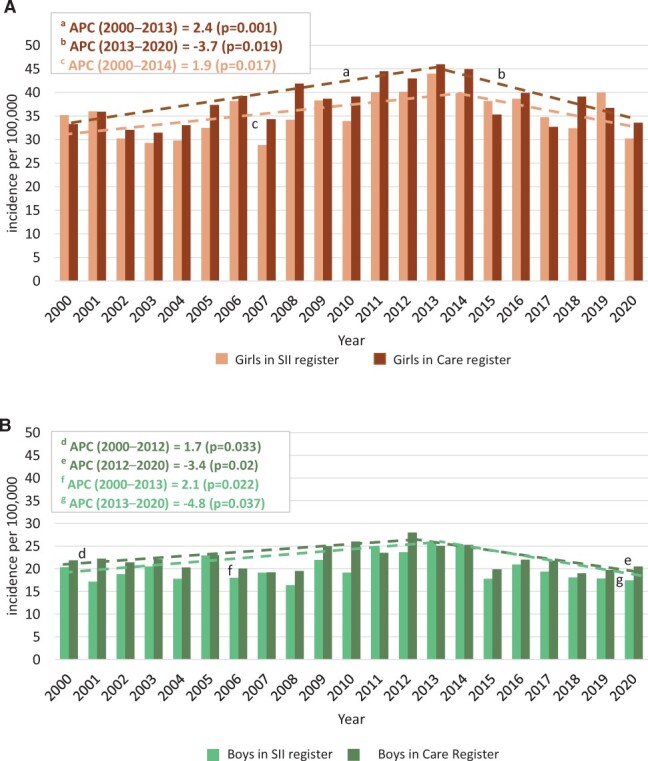
Annual age-adjusted incidence of JIA among children in Finland. Annual age-adjusted incidence rates of JIA per 100 000 children <16 years of age among girls (**A**) and boys (**B**). Joinpoint analyses were performed to detect the best fit model for annual trends in incidence. The figure indicates changes in incidence rates during 2000–2020 in the Care Register of National Institute for Health and Welfare (Care Register) and the Reimbursement Register of the Social Insurance Institution (SII register). Segments (dotted lines) with a statistically significant annual percentage change (APC) are marked a–g

As the birth rate decreased during the follow-up from 56 550–46 524, the age distribution of the population in different years has changed during that time. The age distribution of the population in the year 2000 was used as the standard population to calculate comparable age-adjusted JIA incidences in Fig. 1. Age group incidence rates were reported as crude values.

We used R version 4.3, and the tidyverse collection of R packages for manipulating data and generating plots [20, 21]. Joinpoint analyses were performed in the Joinpoint Regression 4.9.0.1 Program. Standardized normal deviate tests were performed in StatsDirect 3.3.6 statistical software. Two-sided *P* values <0.05 were considered to indicate statistical significance.

## Results

### Clinical characteristics of the study population

A total of 6386 new JIA cases in the Care Register were diagnosed during the study period, 3961 (62.0%) of which were girls. In the SII register, the corresponding numbers were 5556 and 3487 (62.8%), respectively. The mean age at diagnosis was 8.4 years (95% CI 8.3, 8.6) in the Care Register and did not differ from that in the SII register.

### General JIA incidence rates in the study population

**Figure 2.**
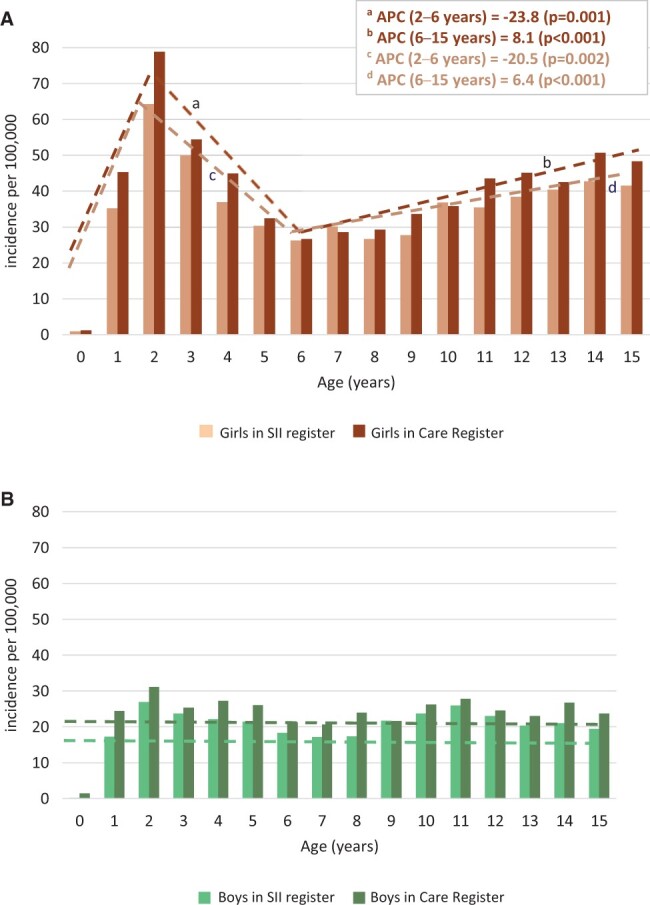
Incidence of JIA in the indicated age groups by sex. Incidence rates in girls (**A**) and boys (**B**) per 100 000 children <16 years for different age groups in 2000–2020 in Finland. Joinpoint analyses were performed to detect the best fit model for age groups. The incidence was stable in boys (**B**) in all groups, whereas in girls (**A**), the incidence peaked at 2 years of age and also increased at 6–15 years of age. Segments (dotted lines) with statistically significant annual percentage change (APC) are marked a–d

**Figure 3.**
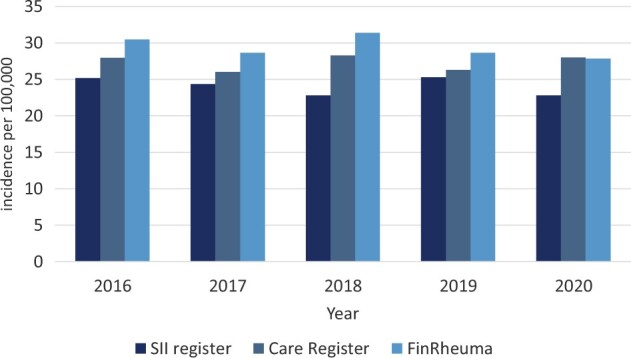
Incidence of JIA in the three study registers in eight hospital districts. Annual incidence rates of JIA from the Care Register of National Institute for Health and Welfare (Care register), the Reimbursement Register of the Social Insurance Institution of Finland (SII register), and the Finnish Rheumatology Quality Register (FinRheuma) in 2016–2020 in eight hospital districts in Finland

The annual incidence rate was 31.7 per 100 000 (95% CI 30.2, 33.1 per 100 000) among all children <16 years of age during the years 2000–2020 in the Care Register (range 28.3–37.6). In the SII register, the corresponding number was 27.5 (95% CI 26.1, 29.0 per 100 000; range 23.6–34.6). Because of the absence of statistically significant differences in incidence rates among registers, the results from the Care Register are reported in the text. The results from the other registers are shown in Figs 1–3.

### Annual incidence of JIA by sex

Girls had higher annual incidence rates than boys during the years 2000–2020. The annual incidence rate was 40.1 per 100 000 for girls and 23.5 per 100 000 for boys during the entire study period.

Joinpoint analyses were performed to detect the best fit model for annual trends in incidence. According to the Care Register, the age-adjusted JIA rate increased by 2.4% per year (95% CI 1.2, 3.7; *P* = 0.001) among girls until 2013, and subsequently decreased by 3.7% per year (95% CI −6.6, −0.7; *P* = 0.019) until 2020 (Fig. 1). Among boys, similar increasing and decreasing age-adjusted incidence rates were observed: +1.7% per year (95% CI 0.1, 3.2; *P* = 0.033) until 2012 and then −3.4% (95% CI −6.0, −0.6; *P* = 0.02) to the end of the study period. When analysing age-adjusted data, there was minimal effect on the results compared with crude data.

### Annual incidence of JIA by age

Two incidence rate peaks were observed at the age of 2 years and in teenagers (Fig. 2). These peaks were due to the high incidence of girls in these age groups. Joinpoint analysis indicated that girls had an incidence peak at 2 years of age, and the incidence subsequently decreased until the age of 6 years, then increased until the age of 15 years (Fig. 2A). The incidence rates were stable in boys at all ages (Fig. 2B). The findings were similar between the Care and SII registers (Fig. 2).

**Figure 4.**
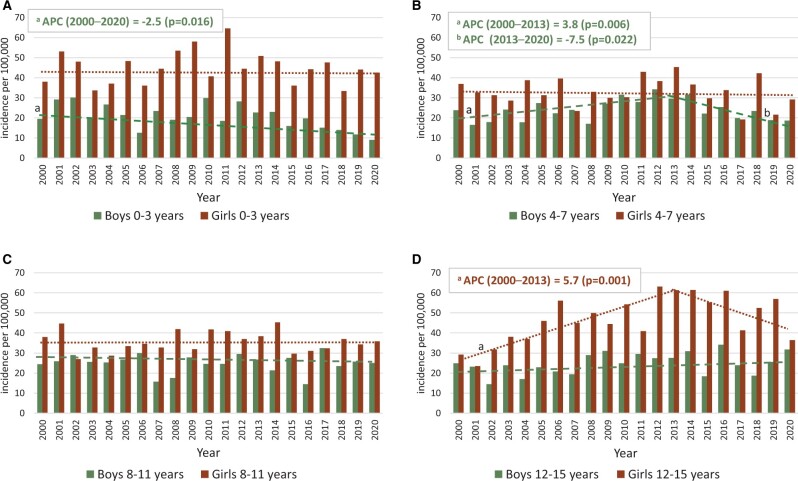
Changes in the incidence of JIA in the indicated age groups. Incidence rates per 100 000 children <16 years of age among boys and girls in different age groups during the study period in the Care Register of National Institute for Health and Welfare. Age groups 0–3 years old (**A**), 4–7 years old (**B**), 8–11 years old (**C**) and 12–15 years old (**D**). Joinpoint analyses were performed to detect the best fit model for annual trends in incidence. The figure shows statistically significant changes among boys 0–3 years old (**A**) and 4–7 years old (**B**), and among girls 12–15 years old (**D**). Segments (green dotted lines for boys and orange for girls) with statistically significant annual percentage change (APC) are marked with a and b

We analysed the changes in the age distribution of new JIA cases during the study period. Among girls, the only significant change in JIA incidence was a 5.7% increase (95% CI 2.8%, 8.6%; *P* = 0.001) among girls 12–15 years old from 2000 to 2013 (Fig. 4). Among boys 0–3 years old, a decreasing incidence was observed during 2000–2020 (APC −2.5%, 95% CI −4.5%, −0.5%; *P* = 0.016). Among boys 4–7 years old, the incidence increased until 2013, then declined, and showed no overall change during the entire study period (Fig. 4). In other age groups no statistically significant differences in incidence were found.

### Regional variations in JIA incidence

**Figure 5.**
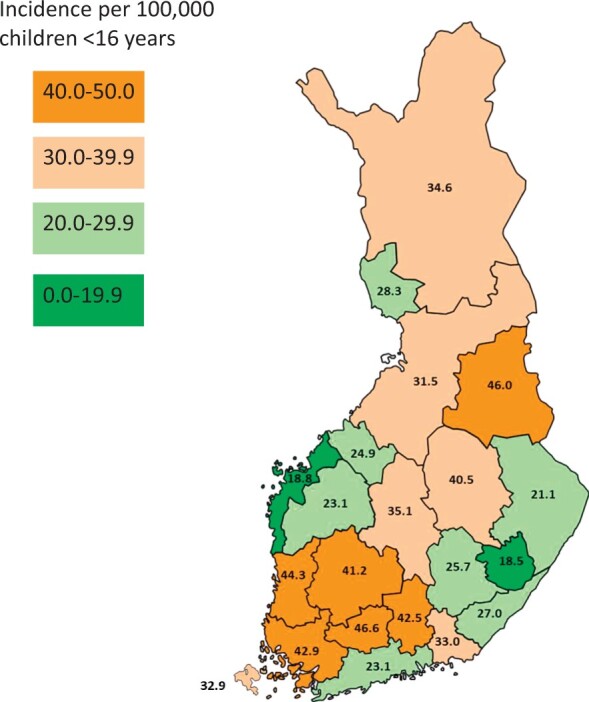
Regional differences in JIA incidence in Finland. Geographical distribution of the mean annual incidence rates of JIA per 100,000 children in all 21 Finnish hospital districts during the years 2000–2020. Data are from the Care Register of National Institute for Health and Welfare

Geographical differences in the incidence of JIA in Finland were observed across the 21 hospital districts during the years 2000–2020. The rates varied from 18.5 per 100 000 (Eastern Savonia) to 46.6 per 100 000 (Tavastia Proper) and showed no specific geographical patterns in Finland (Fig. 5).

### FinRheuma register and distribution of JIA categories

A total of 887 new JIA cases were diagnosed during the years 2016–2020 in the eight hospital districts, according to the FinRheuma register. The annual incidence rate was 29.4 per 100 000 among children <16 years of age, a value slightly higher but in line with the rates from the two other registers for the same time periods and regions (Fig. 3): 27.3 per 100 000 in the Care Register and 24.1 per 100 000 in the SII register. The distribution of JIA categories was 45.1% for oligoarthritis (M08.4) (persistent 43.3%, extended 1.8%), 24.1% for RF− polyarthritis (M08.3), 11.2% for enthesitis-related arthritis (M08.1), 6.1% for newly diagnosed yet unspecified (M08.9), 4.6% for unknown category (M08), 3.6% for psoriatic arthritis (M09.0*L40.5), 2.5% for systemic JIA (M08.2), 1.8% for RF+ polyarthritis (M08.0) and 1.0% for unclassified JIA (M08.8).

## Discussion

In this long-term population study based on data from three comprehensive national registers, we found that the incidence of JIA was 27.5–31.7 per 100 000 per year. This was higher than those previously reported both in Finland and globally. The incidence peaked in the years 2010–2013 but did not increase from 2000 to 2020. Regional differences in incidence were observed across hospital districts.

Our rates are higher than those in previous epidemiological studies of JIA in Finland, in which the incidence rates have been reported to increase from 14 per 100 000 [16] to 23 per 100 000 [18]. Several differences in study design might explain these differences. Previous studies have examined limited time periods consisting of only a few consecutive years [13, 18, 19] and have covered geographically restricted areas of Finland [13, 16–19]. In contrast, our study used regional data from multiple registers over two decades, covering the entire country.

The incidence of JIA is high in Finland with respect to other Scandinavian and European countries. The reported rates have varied from 12.8 to 17.1 per 100 000 in Sweden [13, 14, 22] and from 14 to 23 per 100 000 in Norway [13, 23]. In Southern Europe, the rates have ranged from 3.2 [7] to 6.9 per 100 000 [9] and have been reported to be 5.6 per 100 000 in the UK [8]. The study periods were shorter in earlier studies [9, 13]. Moreover, some studies have used previous juvenile arthritis criteria instead of ILAR [13] and have not focused on only JIA [23]. In Germany, Horneff *et al.* have reported an incidence rate of JIA as high as 34–60 per 100 000 among children 2–15 years old, according to data from two databases [24]. However, they also included the ICD-10 code M13 (other arthritis), which can describe any short-term arthritis, and extrapolated the results to the entire population. Moreover, they did not include children less than 2 years old, in whom the incidence is low. The differences in incidence rates across studies might be due to differences in study designs and periods, data collection, JIA classification criteria and geographical region selection. In Finland, JIA is diagnosed and treated in special paediatric rheumatology clinics in hospitals, where a physician must register a patient’s diagnosis code in the Care Register at all visits, and registration in the FinRheuma register is mainly a routine in most hospitals. Therefore, our findings regarding patients with JIA are likely to be based on comprehensive data. In countries without accurate registers, not all JIA cases may be reported.

Although we observed annual variations in the incidence of JIA, it remained relatively stable during the years 2000–2020. To our knowledge, only one previous long-term study has assessed the incidence of JIA in Finland and has reported an increase in incidence from 14 to 19.5 per 100 000 from the 1980s to 1995 [17]. However, that study included only 11 of 21 hospital districts, and used the American College of Rheumatology (ACR) instead of ILAR criteria. Since then, diagnostic methods have improved, and use of magnetic resonance imaging (MRI) has become more common, thus potentially partially accounting for the increased number of cases. JIA incidence has also been speculated to be cyclical [12, 25, 26] and long-term studies are necessary to determine whether the annual fluctuation observed in our study might be part of a cyclical pattern. Advanced diagnostic tools, regional changes in care responsibilities following the closure of the rheumatology hospital in Heinola, infections and vaccines could be reasons for changes in incidence across different age groups (Fig. 4).

The COVID-19 pandemic started in Finland at the beginning of 2020, when the incidence of JIA was simultaneously decreasing, according to our results. Changes in the incidence of other autoimmune diseases have been observed after the onset of the pandemic, but information regarding this phenomenon in children is still limited [27, 28]. Future studies are necessary to assess whether an association might exist between JIA aetiology and viral infections, or whether the lower incidence during the pandemic has been due to avoidance of seeking medical attention to limit social contact during the pandemic.

Globally, conflicting results have been reported regarding epidemiological changes in JIA. A steady incidence with temporal variation, as found in our study, has been observed in previous long-term studies in Denmark [15] and in the USA [12]. A recently published Swedish study [22] has reported an increasing incidence of JIA, as has an Italian study [29]. However, those studies were limited to the time before 2010 [22] or included only limited areas of the country [29].

The predominance of JIA in girls is well known, both in Finland [16] and globally [2, 12–15, 24, 29], but has not been observed in the Taiwanese population [30]. In our study, the ratio of girls to boys with JIA was 1.7:1 and did not significantly change during the study period, in agreement with findings from several previous studies [2]. Horneff *et al.* have reported an increase in the ratio of girls to boys with JIA with increasing age in Germany [24]. Similar biphasic incidence peaks for girls and stable incidence for boys of different ages have been reported in previous Nordic studies [13, 15, 31]. A peak in the incidence of JIA has been observed for boys 12 years of age [14].

We observed significant geographical differences in JIA incidence, but we did not find specific south-north differences, as previously speculated to exist in European studies [13]. In our study differences in recording methods and coverage of the registers as well as regional over- and underdiagnosis cannot be excluded. Our results support previously reported estimates regarding the roles of genetic factors in the aetiology of JIA [32, 33]. One of the lowest incidences was observed in Ostrobothnia, which had a rate similar to that previously reported in Sweden [13, 14], a geographical neighbouring country with a shared genetic background. Regional differences in Finland were also reported in the 1980–1990s, although those studies covered only limited areas [17]. During the years 1946–2010, a special rheumatology hospital in Heinola in Finland had an important role in the diagnosis and treatment of JIA. After 2010, the diagnosis of JIA has been geographically decentralized in Finland, thus potentially leading to diagnostic heterogeneity. This factor might have influenced the regional differences in incidence rates. In addition, the use of MRI can vary regionally, and the interpretation of paediatric imaging findings is challenging and partly dependent on the radiologist.

Moreover, geographical differences in the incidence of other chronic autoimmune diseases, such as type I diabetes [34] and multiple sclerosis [35, 36], have been observed, thus potentially indicating that common genetic factors may underlie these diseases. Further studies are needed to understand the role of genetics in the aetiology of JIA.

## Strengths and limitations

This study has several strengths. This was a nationwide population study based on long-term data for all regions in Finland. The incidence rates were consistent among all three registers, and the FinRheuma data were separately confirmed, based on patient medical records. Because Finland has a universal healthcare system, and nearly all JIA cases are treated in public hospitals, at paediatric or paediatric rheumatology clinics, selection bias was diminished. At these clinics registry reporting is usually routine. Moreover, use of MRI has become more common, and its accuracy has improved, thus increasing the sensitivity of arthritis diagnosis, particularly sacroiliitis and temporomandibular joint inflammation, which are otherwise difficult to diagnose. Therefore, it is likely that patients of all ages and categories are registered in Finland, and that the incidence rates, reported here, are reasonably specific.

This study also has limitations. This was a retrospective register study and, therefore, subjected to the general weaknesses inherent in this kind of study design. Data analysis from clinical registers always includes a risk of over- and underdiagnosis. By combining the results from three registers, we were able to accurately estimate the incidence rates. The SII register contains patients approved for reimbursement for JIA treatments. Therefore, mild cases not needing DMARD treatment are not registered in the SII database, and the register indicates the minimum incidence. In contrast, the Care Register might slightly overestimate the number of new JIA cases. A potential small risk of misclassification of some cases as JIA rather than, for example, short-term transient reactive arthritis, might possibly have led to overestimation of the incidence. FinRheuma includes data from several hospital districts, where several physicians fill out the register; therefore, diagnosing and subtyping can be heterogeneous in register studies. In contrast, data from eight hospital districts, where the data had been separately verified for possible errors, were included from the FinRheuma register.

## Conclusion

The incidence of JIA is very high with respect to that in other parts of the world and is higher than previously reported in Finland. We observed a peak in incidence in the years 2010–2013, but the incidence was not higher at the end of our study interval, in the years 2019 and 2020, than at the beginning, in the year 2000. Decreasing incidence was observed among boys 0–3 years old during the entire study period, and increasing incidence was observed among teenage girls and boys 4–7 years old from 2000 to 2013. Further studies are needed to clarify the potential cyclicality of JIA incidence.

## Data Availability

Deidentified individual participant data will be made available, in addition to study protocols and the statistical analysis plan. The data will be made available upon publication until February 2034 to researchers who provide a methodologically sound proposal for use in achieving the goals of the approved proposal. Proposals should be submitted to maria.backstrom@ovph.fi.
